# Supplementary material for: Remimazolam’s clinical application and safety: A signal detection analysis based on FAERS data and literature support
Source: PLoS One. 2025 Aug 22;20(8):e0330769. doi: 10.1371/journal.pone.0330769 (PMC12373250; doi:10.1371/journal.pone.0330769)
Supplement: Attachment — Submitted filename: PONE-D-24-58735_Response_to_Reviewers.docx [file pone.0330769.s003.docx]

Dear Dr. Anmar Al-Taie,
Academic Editor,
PLOS ONE

Thank you for your careful consideration of our manuscript, “Remimazolam’s Clinical Application and Safety: A Signal Detection Analysis Based on FAERS Data and Literature Support” (PONE-D-24-58735), and for the constructive feedback from both yourself and the reviewer.

Please accept our sincere apologies for the time taken to submit this revision. We wanted to ensure that every point was addressed with the thoroughness and care it deserved.

We have now completed all revisions based on the feedback and believe the manuscript has been significantly improved as a result. We found all comments to be extremely helpful, and below, we provide a point-by-point response to all requirements.

**Part I: Response to the Journal Requirements from the Academic Editor**

**Requirement 1: "** Please ensure that your manuscript meets PLOS ONE's style requirements, including those for file naming. The PLOS ONE style templates can be found at https://journals.plos.org/plosone/s/file?id=wjVg/PLOSOne_formatting_sample_main_body.pdf and https://journals.plos.org/plosone/s/file?id=ba62/PLOSOne_formatting_sample_title_authors_affiliations.pdf**"**

**Response:** Thank you for this important reminder. We have carefully gone through the entire manuscript and cross-referenced it with the PLOS ONE style templates. We have made sure that all aspects, from file naming and headings to the formatting of figures, tables, and the reference list, are now fully compliant with the journal's requirements.

**Requirement 2:** " Please note that PLOS ONE has specific guidelines on code sharing for submissions in which author-generated code underpins the findings in the manuscript. In these cases, we expect all author-generated code to be made available without restrictions upon publication of the work. Please review our guidelines at https://journals.plos.org/plosone/s/materials-and-software-sharing#loc-sharing-code and ensure that your code is shared in a way that follows best practice and facilitates reproducibility and reuse."

**Response:** We wholeheartedly agree with the journal's commitment to reproducibility and open science. We have reviewed the guidelines and are pleased to share our complete, author-generated R script which underpins our findings. It has been uploaded as a Supporting Information file (S1_File.R) and clearly described in the manuscript to facilitate reuse and verification.

**Requirement 3:** "Please ensure that you refer to Figure 1 and 2 in your text as, if accepted, production will need this reference to link the reader to the figure."

**Response:** Thank you for pointing this out. We understand the importance of correct linking for the final production. We have carefully checked the manuscript and can confirm that both Figure 1 and Figure 2 are now explicitly and correctly referenced in the text where they are discussed.

**Requirement 4:** "Please include your tables as part of your main manuscript and remove the individual files. Please note that supplementary tables (should remain/ be uploaded) as separate 'supporting information' files."

**Response:** We appreciate the clear instruction on this. Following your guidance, we have now embedded all main tables (Table 1, 2, and 3) directly into the manuscript file. They are placed immediately after the paragraphs where they are first cited, and the separate table files have been removed from our submission package.

**Requirement 5:** " Please review your reference list to ensure that it is complete and correct. If you have cited papers that have been retracted, please include the rationale for doing so in the manuscript text, or remove these references and replace them with relevant current references. Any changes to the reference list should be mentioned in the rebuttal letter that accompanies your revised manuscript. If you need to cite a retracted article, indicate the article’s retracted status in the References list and also include a citation and full reference for the retraction notice."

**Response:** Thank you. Ensuring the integrity of the reference list is crucial. We have performed a comprehensive review of every reference to verify its accuracy, completeness, and formatting according to the journal's style. We also specifically checked for any retractions and can confirm that all cited papers are current and appropriate.

**Part II: Response to the Comments from Reviewer #1**

We sincerely thank Reviewer #1 for their positive evaluation and insightful suggestions, which have helped us improve the quality of our manuscript.

**Point-by-point response**

Reviewer #1:

Comment

Zhou et al entitle “Remimazolam’s Clinical Application and Safety: A Signal Detection Analysis Based on FAERS Data and Literature Support ” clearly reported that Remimazolam could be adverse drug for procedures requiring quick recovery, procedural sedation, and general anesthesia.

Why are you using the FAERS database to study this drug, it should be that someone else has done this with this similar database similar methodology, so you can write cite in the INTRODUCTION section about some specific other similar studies, such as recommending a few (It is equivalent to saying that someone else has done this type of research using the FAERS database, and you can use this database to do research related to Remimazolam as well):

【1】Wang Y, Zhao B, Yang H, Wan Z. A real-world pharmacovigilance study of FDA adverse event reporting system events for sildenafil. Andrology. 2024 May;12(4):785-792. doi: 10.1111/andr.13533. Epub 2023 Sep 19. PMID: 37724699.

【2】Zhao B, Fu Y, Cui S, Chen X, Liu S, Luo L. A real-world disproportionality analysis of Everolimus: data mining of the public version of FDA adverse event reporting system. Front Pharmacol. 2024 Mar 12;15:1333662. doi: 10.3389/fphar.2024.1333662. PMID: 38533254; PMCID: PMC10964017.

【3】Yang H, Wan Z, Chen M, Zhang X, Cui W, Zhao B. A real-world data analysis of topotecan in the FDA Adverse Event Reporting System (FAERS) database. Expert Opin Drug Metab Toxicol. 2023 Apr;19(4):217-223. doi: 10.1080/17425255.2023.2219390. Epub 2023 May 30. PMID: 37243615.

【4】Zhao B, Zhang X, Chen M, Wang Y. A real-world data analysis of acetylsalicylic acid in FDA Adverse Event Reporting System (FAERS) database. Expert Opin Drug Metab Toxicol. 2023 Jan-Jun;19(6):381-387. doi: 10.1080/17425255.2023.2235267. Epub 2023 Jul 12. PMID: 37421631.

【5】Li, Jie, Zhao, Bin, Zhu, YongQing, Wu, Jibiao, Vitreoretinal Traction Syndrome, Nitrituria and Human Epidermal Growth Factor Receptor Negative Might Occur in the Aromatase-Inhibitor Anastrozole Treatment, International Journal of Clinical Practice, 2024, 5132916, 9 pages, 2024. https://doi.org/10.1155/2024/5132916

【6】Zhong, C., Zheng, Q., Zhao, B., & Ren, T. (2024). A real-world pharmacovigilance study using disproportionality analysis of United States Food and Drug Administration Adverse Event Reporting System events for vinca alkaloids: comparing vinorelbine and Vincristine. Expert Opinion on Drug Safety, 23(11), 1427–1437. https://doi.org/10.1080/14740338.2024.2410436

**Response:** We sincerely thank the reviewer for this insightful comment and for providing a valuable list of relevant literature. We agree that highlighting previous studies employing similar methodologies with the FAERS database strengthens the rationale for our approach.

In response to this suggestion, we have revised the Introduction section to explicitly state why the FAERS database is a suitable and established tool for pharmacovigilance studies like ours. We have now incorporated citations to several studies, including those kindly recommended by the reviewer, that have successfully utilized the FAERS database for signal detection and real-world safety assessments of various drugs.

Specifically, the following paragraph has been added to the Introduction:

"*Indeed, pharmacovigilance studies using real-world databases are instrumental for assessing the post-marketing safety profiles of various therapeutics. These studies complement findings from pre-approval clinical trials, as seen in studies on sildenafil [6] and everolimus [7]. The utility of FAERS for such pharmacovigilance, and its capacity for signal detection in diverse therapeutic areas, is well-documented in studies concerning topotecan [10], acetylsalicylic acid [11], anastrozole [12], and vinca alkaloids [13]*."

We believe these additions clearly demonstrate the precedent and justification for using the FAERS database for our investigation of remimazolam and appropriately acknowledge the utility of this approach as established by prior research. We are grateful for the reviewer's guidance in enhancing this aspect of our manuscript.

Minior Issues:

**Question1:** Simplify complex sentences to improve readability. For example, revise "particularly focusing on its safety in the respiratory, cardiovascular, and immune systems" to "with a focus on its safety in major organ systems, including respiratory, cardiovascular, and immune systems.

Response: Thank you for this essential feedback. We are sincerely grateful for your specific guidance on improving the manuscript's clarity, as this is a point of utmost importance to us.

We would also like to transparently explain our revision process in response to your invaluable feedback. Upon receiving your comments, we first addressed this specific point by performing a thorough revision of the entire manuscript based on the principle you outlined.

Subsequently, your other linguistic feedback (particularly in Point 6) inspired us to undertake an even deeper, more holistic overhaul of the paper's language. This second-stage revision was so comprehensive that many sections, including the one containing the original example sentence, were completely rewritten to improve flow and impact.

Therefore, while the exact sentence you highlighted may no longer exist in its revised form, please be assured that the core principle of your suggestion was the guiding force behind our entire linguistic refinement. To demonstrate our commitment to your advice, here is the methodology we applied systematically across the whole manuscript:

We broke down long, complex sentences into shorter, more direct statements.

We rephrased complex clauses and participial phrases to make logical connections explicit and unambiguous.

We used simpler conjunctions and clearer transitions to guide the reader smoothly through our arguments.

We hope this explanation clarifies our dedicated approach. Your combined feedback prompted a level of revision that we believe has elevated the entire manuscript. We are truly appreciative of the opportunity you gave us to so thoroughly improve our work.

**Question2**: Clarify the time frame covered by the FAERS data (e.g., specific months or quarters) to enhance transparency.

**Response:** Thank you for highlighting the need for greater transparency on this point. To address this comprehensively, we have revised the "Data Source and Preprocessing" subsection in our Methods. We now explicitly state that our search encompassed the full database period from 2004 to 2023, and clarify that the resulting reports for our drug of interest, Remimazolam, were found to span from 2020 to 2023. This detail is crucial for accurately describing our dataset. The revised text in the manuscript now reads:

*"* *This study is based on data from the U.S. Food and Drug Administration's (FDA) Adverse Event Reporting System (FAERS), a public database of spontaneous adverse event reports submitted by patients, healthcare professionals, and pharmaceutical manufacturers. For our analysis, we queried the entire available database, spanning from the first quarter of 2004 to the fourth quarter of 2023, to extract all reports associated with Remimazolam. The resulting dataset of relevant reports for this drug covered the period from the second quarter of 2020 to the first quarter of 2023, and this forms the basis of our study."*

**Question3:** Explain why a threshold of three cases was chosen for signal analysis in "Signal Screening and Classification.".

**Response:** Thank you for this insightful question and the opportunity to elaborate on this key methodological choice.

Our selection of a minimum of three reports (N ≥ 3) is a standard pharmacovigilance practice designed to ensure the statistical robustness of our findings. This threshold serves two primary functions:

1. **Enhancing Statistical Stability:** It provides a more stable foundation for disproportionality calculations (like ROR), reducing the risk of spurious signals that can arise from one or two isolated reports.
2. **Reducing Analytical Noise:** It helps filter out coincidental or isolated case reports common in spontaneous reporting systems, thereby improving the specificity of the detected signals.

This approach is well-supported in the literature. For instance, a study by Lerch et al. (2015) in the journal *Drug Safety* employed this exact criterion (N ≥ 3) for signal detection in the FAERS database.

To make this rationale clear in the manuscript, we have now added the following explanation to the *Methodology* section, under the *Signal Screening and Classification* subsection, and have included the supporting citation:

"*This threshold (N ≥ 3) is a commonly adopted criterion in pharmacovigilance to enhance the stability of disproportionality measures and to reduce the likelihood of spurious signals arising from isolated case reports [14]*."

We thank you for prompting this important clarification.

**Question4:** For Table 2, provide a brief interpretation of the ROR and PRR values in the text, especially for high-risk signals like hypoventilation and anaphylactic shock, to aid readers unfamiliar with these metrics..

**Response:** Thank you for this excellent suggestion. We agree that providing a clear interpretation of the ROR values is essential for making our findings accessible to a broader audience.

To address this, we have substantially revised the *Results* section. **Within the main text**, for each of the key adverse events discussed, we have now added a plain-language interpretation of its corresponding ROR value from Table 2.

For instance, for the high-risk signals you highlighted:

- For **Hypoventilation**, which has an ROR of approximately 450, we now explain in the text that this means the event was reported over 400 times more frequently than expected.
- For **Anaphylactic shock**, with an ROR of about 106, we clarify in the text that reports were more than 100 times more frequent than expected.

This interpretive approach has been applied systematically throughout the text to other significant signals, such as **Cardio-respiratory arrest** and **Blood pressure decreased**, to give readers a clear sense of the signal strength.

We are confident that these additions make the clinical implications of our quantitative findings much clearer, and we appreciate your guidance on improving the paper's accessibility.

**Question5:** Improve Figure 1's clarity by enlarging text and refining the labels for better visualization.

Response: Thank you very much for your thoughtful comment. I have made sincere efforts to improve the clarity of Figure 1, including enlarging the text and refining the labels as suggested. However, due to limitations in PDF rendering, some loss of resolution may still occur in the final compiled version.

To address this, I have re-inserted a high-resolution version of the figure directly into the main manuscript, following the editorial office's instruction to embed all figures within the text. While the clarity has been improved, the figure might still appear slightly different compared to earlier versions.

The revised figure is placed below for your kind review. Should you still find the clarity unsatisfactory, please kindly indicate it in your response, and I will make further revisions accordingly.

**Question6:** Correct minor grammatical issues, such as replacing "remimazolam may trigger serious immune responses in clinical use" with "remimazolam may elicit serious immune responses in clinical settings."

Response:

Thank you for your invaluable guidance. The phrasing you suggested is indeed far more precise and professional.

Following your expert advice, we have not only incorporated this specific change but have also taken this opportunity to conduct a comprehensive review of the entire manuscript. We have meticulously polished the language throughout the paper to enhance its overall clarity, precision, and scholarly tone.

We are sincerely grateful for the chance to improve our work under your direction. Your keen insights have been instrumental in elevating the quality of our manuscript to meet the high standards of PLOS ONE.

**Question7:** Include a forward-looking statement emphasizing the need for multicenter prospective studies to validate these findings.

Response:

Thank you for this important suggestion. We agree that adding this forward-looking statement strengthens the manuscript's conclusion.

Following your advice, we have revised the final paragraph of our Conclusion section to explicitly emphasize the need for future validation. The revised text now includes the statement: "*To advance from signal detection to confirmed risk assessment, it is essential that these findings are validated through large-scale, multicenter prospective studies.*"

We believe this revision improves the paper, and we thank you again for your valuable input.

We thank you and the reviewer once again for your valuable time and guidance. We have uploaded a clean version of the manuscript and a version with tracked changes for your convenience. We hope that the revised manuscript is now suitable for publication in *PLOS ONE*.

Sincerely,

Qingbo Zhou, on behalf of all authors.

**Dr. Qingbo Zhou**
Department of Internal Medicine, Shaoxing Yuecheng People's Hospital
Shaoxing, China
**E-mail:** [zhouqingbo@zcmu.edu.cn](mailto:zhouqingbo@zcmu.edu.cn)

**(Co-authors: Gang Ye¹, Luqin Ding²)**
¹ Department of Pain Medicine, Shaoxing People's Hospital, Shaoxing, China
² Department of Internal Medicine, Shaoxing Yuecheng People's Hospital, Shaoxing, China
