# Supplementary material for: Remimazolam’s clinical application and safety: A signal detection analysis based on FAERS data and literature support
Source: PLoS One. 2025 Aug 22;20(8):e0330769. doi: 10.1371/journal.pone.0330769 (PMC12373250; doi:10.1371/journal.pone.0330769)
Supplement: Attachment — Submitted filename: Response to Editor.docx [file pone.0330769.s004.docx]

July 30, 2025

Dr. Anmar AL-TAIE
Academic Editor
PLOS ONE

**Subject: Submission of Revised Manuscript (ID: PONE-D-24-58735R1)**

Dear Dr. AL-TAIE,

Thank you for your email and for the opportunity to revise our manuscript, “Remimazolam’s Clinical Application and Safety: A Signal Detection Analysis Based on FAERS Data and Literature Support” (ID: PONE-D-24-58735R1). We sincerely appreciate the time and effort you have dedicated to providing feedback on our work.

We have carefully considered all the comments and have revised the manuscript accordingly. We believe the manuscript has been significantly improved as a result. Below is a point-by-point response to the comments.

**Response to Editor's Comments:**

**Comment 1: "There are some minor grammatical issues that need to be corrected."**

**Our Response:** We thank you for this valuable feedback. We have thoroughly proofread the entire manuscript to correct all grammatical and stylistic issues. We have carefully revised sentence structures, ensured consistent use of verb tenses, improved punctuation, and enhanced the overall clarity and readability of the text. We are confident that the revised manuscript now meets the journal's standards for language.

**Comment 2: "What are the authors trying to say by this incomplete sentence’ reduce the probability of spurious signals from isolated case reports (e.g.,[19]).’’”**

**Our Response:** We sincerely apologize for this unclear sentence structure and thank you for pointing it out. The original phrasing was awkward. We intended to explain the rationale for setting a minimum threshold for adverse event reports.

We have now revised this sentence in the **“Signal Screening and Classification”** section to be clear and complete. The sentence now reads:

"This threshold is a standard practice in pharmacovigilance to increase the stability of the analysis and reduce the probability of spurious signals from isolated case reports [19]."

**Comment 3: "The text for some responses is not available within the main paper body. Try to be committed to the responses."**

**Our Response:** We would like to offer our sincerest apologies for this critical oversight and the inconsistency between our previous rebuttal letter and the revised manuscript. This was our mistake, and we deeply appreciate you bringing it to our attention.

We have now taken corrective action to ensure all promised changes are implemented. The sentence regarding the methodological justification for the N ≥ 3 threshold, which we had previously promised to include, has now been correctly inserted into the **“Signal Screening and Classification”** section of the manuscript.

To ensure full compliance, we have conducted an additional, thorough review of the entire manuscript against our previous rebuttal letter to confirm that all promised changes have been implemented accurately. We apologize again for the inconvenience this has caused.

**Comment 4: "The authors mention several times ‘such as elderly patients and individuals with pre-existing comorbidities’’ We could not recognize these comorbidities with this small sample size. In addition, there are no results that show such an association with these variables. Consider addressing this concern while providing more details and a table for the common comorbidities."**

**Our Response:** We are exceptionally grateful for this insightful and critical comment. We agree completely that our previous statements regarding high-risk populations were not supported by our own data. This was a significant oversight, and we sincerely apologize. Your feedback has been invaluable in helping us correct this, thereby greatly enhancing the scientific integrity and credibility of our manuscript.

As you suggested, we initially considered creating a table for comorbidities. However, upon careful re-evaluation of our small dataset (N=68), we concluded that the available comorbidity data was not sufficiently detailed or robust for a meaningful analysis. We believe that presenting a table based on such limited information could be potentially misleading to readers.

Therefore, to address your concern in the most scientifically rigorous manner, we have undertaken a comprehensive revision of the **Abstract, Discussion, and Conclusion** sections. We have meticulously rephrased every statement that linked adverse event risks to specific populations (such as the elderly or those with comorbidities). These statements now clearly and explicitly attribute such information to the existing body of literature, using it as context for our findings, rather than presenting it as a conclusion from our analysis.

For example, a statement in the Discussion section was revised from:

- **Original:** "These risks appear to be most pronounced in high-risk populations, such as elderly patients and individuals with pre-existing comorbidities[3]."
- **Revised:** "Existing literature suggests that such risks can be more pronounced in high-risk populations, such as elderly patients and individuals with pre-existing comorbidities [3], which underscores the need for heightened caution..."

We believe these thorough revisions have fully resolved the issue by ensuring our conclusions are strictly aligned with our data. We are truly thankful for your guidance, which has prevented us from making a serious error and has significantly improved the quality of our paper.

We hope that the revised manuscript is now suitable for publication in *PLOS ONE*. We look forward to hearing from you.

Sincerely,

Dr. Qingbo Zhou
Corresponding Author
Department of Internal Medicine, Shaoxing Yuecheng People's Hospital, Shaoxing, China
E-mail: [zhouqingbo@zcmu.edu.cn](mailto:zhouqingbo@zcmu.edu.cn)
